# Red carpet moments: recognition of neuroscientists by election to UK national academies

**DOI:** 10.1093/braincomms/fcae203

**Published:** 2024-07-03

**Authors:** Tara L Spires-Jones

**Affiliations:** Edinburgh, UK

## Abstract

Our editor discusses recognition of achievement in translational neuroscience and the wider issues around incentivization of research.

## Graphical Abstract

**Graphical Abstract:**
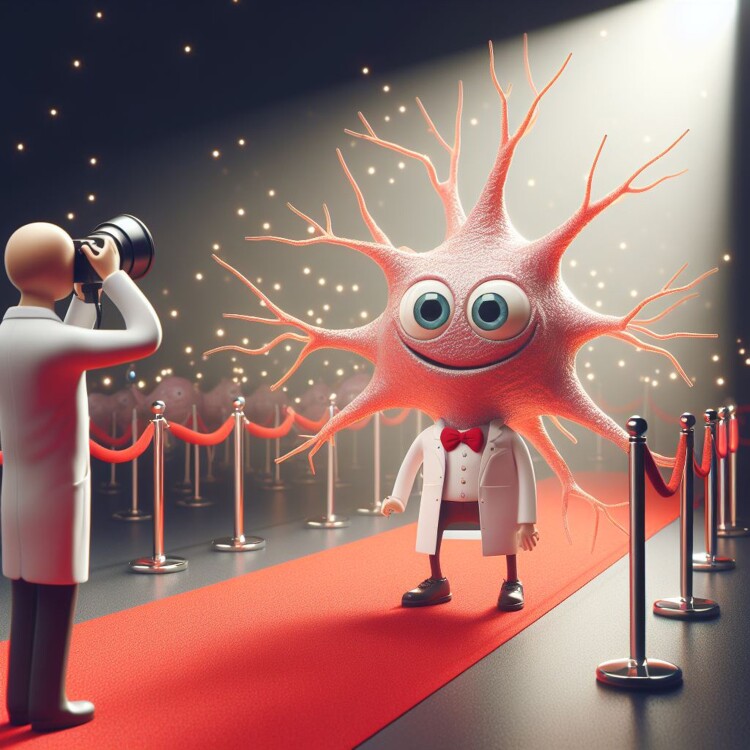

Welcome to Volume 6, issue 4 of *Brain Communications*. In the week when I’m writing this editorial, new 2024 Fellows of the Royal Society and Fellows of the Academy of Medical Sciences have been announced in the UK. These learned academies promote science and its benefits and recognize excellence in scientific research. I am honoured to be one of the new Fellows of the Academy of Medical Sciences and delighted to see so many neuroscientists and indeed *Brain Communications* authors honoured in these elections.

While there are too many eminent neuroscientists among the newly elected fellows to cover all of them, new Fellows of the Royal Society include Prof. Sarah Tabrizi, one of the Guarantors of *Brain*, who has co-authored six papers in our journal including important negative results on lack of improvement of neuroinflammation in a Huntington’s disease clinical trial.^1^ Prof. Ammar Al-Chalabi, another Guarantor and Associate Editor of *Brain*, was elected as a Fellow of the Academy of Medical Sciences (FMedSci), and has co-authored five papers in *Brain Communications* including work on microRNA involvement in life expectancy of people with amyotrophic lateral sclerosis.^2^ Prof. James Boardman, another newly elected FMedSci, from my neck of the woods in Edinburgh, published a study with us in 2022 examining DNA methylation in preterm infants.^3^ Prof. Rustam Al-Shahi Salman, FMedSci, was co-author of a *Brain Communications* paper examining risk factors for neurological and psychiatric complications of COVID-19 in a nationwide surveillance study.^4^

As well as honouring scientists through the election of fellows, learned societies play an important role in the scientific ecosystem. Both the Royal Society and the Academy of Medical Sciences are national academies that represent the UK research community and advocate for science and its benefits. The Royal Society, founded in 1660, is the oldest continuously existing scientific academy in the world and one of the pioneers of disseminating scientific findings through publication.^5^ The Academy of Medical Sciences was founded in 1998 as one of four UK national academies with the intention of creating a national resource, independent of the government, bringing together biomedical scientists and academics to promote translation of medical science into benefits for society. Prof. Sir John Savill, commenting in 1999 on the formation of the Academy of Medical Sciences, was hopeful that the academy would improve research culture in academic medicine calling the academy ‘a good thing’.^6^ As independent expert bodies, these societies are often consulted by the government on important scientific issues. The academies also offer grants and do important work to support research culture.

However, as with any institution, the academies are not perfect. Unlike the British Neuroscience Association, Society for Neuroscience and other membership organizations that can be joined by any neuroscientist, by design, the fellowship of the academies is elitist, recognizing the ‘best of the best’. Over the years, the national academies have been criticized for this elitism and for not being representative of the full diversity of scientists. One example of this is that it was 285 years from its founding before the Royal Society admitted its first women, Kathleen Lonsdale and Marjory Stephenson. The academies are in a way ‘closed shops’ since new members are elected by existing members, adding to their reputations as old boys’ networks. However, there is evidence that the academies are working towards inclusion both in their memberships and their wider activities like grants (https://acmedsci.ac.uk/about/ourwork/equity-diversity-and-inclusion/edi-strategy  https://royalsociety.org/current-topics/diversity/).

Another potential downside of the academies for fellows was highlighted by my teenage son. When I got the news of being elected as FMedSci, I texted the family, very excited about the recognition and the prospect of more letters after my name. My son’s response was ‘Will this mean any more work for you? Will you have to take more work trips? Do you get any more money for this?’ The answers were of course yes, yes and no as fellows work for the academy in many ways and are not paid for this charitable work. Adding to the amount of service work not related to core research and teaching activities is daunting. Like many of you, I’m buried in administrative tasks as well as reviewing papers and grants. While the ‘reward’ of recognition is very nice, it raises the wider question of incentives and recognition in neuroscience and how the academies might contribute to this wider issue of research culture.

On balance as a new ‘good old boy’, I believe that the national academies are a force for good, and I look forward to working with other fellows to improve both research culture and evidence-based policy.

The cover image for this issue shows a graphical representation of the conceptual activities of small non-coding regulatory RNAs, which have emerged as contributing to the loss of personal cognitive memories in the brain of some, but not all Alzheimer’s disease patients.^7^ Image courtesy of Dr Eyal Soreq.
